# Directed Evolution of Aminoglycoside Phosphotransferase (3′) Type IIIa Variants That Inactivate Amikacin but Impose Significant Fitness Costs

**DOI:** 10.1371/journal.pone.0076687

**Published:** 2013-10-18

**Authors:** Joseph R. Kramer, Ichiro Matsumura

**Affiliations:** Emory University School of Medicine, Department of Biochemistry, Rollins Research Center, Atlanta, Georgia, United States of America; The Scripps Research Institute, United States of America

## Abstract

The rules that govern adaptive protein evolution remain incompletely understood. Aminoglycoside aminotransferase (3′) type IIIa (hereafter abbreviated APH(3′)-IIIa) is a good model enzyme because it inactivates kanamycin efficiently; it recognizes other aminoglycoside antibiotics, including amikacin, but not nearly as well. Here we direct the evolution of APH(3′)-IIIa variants with increased activity against amikacin. After four rounds of random mutation and selection in *Escherichia coli*, the minimum inhibitory concentration of amikacin rose from 18 micrograms/mL (wild-type enzyme) to over 1200 micrograms/mL (clone 4.1). The artificially evolved 4.1 APH(3′)-IIIa variant exhibited 19-fold greater catalytic efficiency (*k*_cat_/*K*_M_) than did the wild-type enzyme in reactions with amikacin. *E. coli* expressing the evolved 4.1 APH(3′)-IIIa also exhibited a four-fold decrease in fitness (as measured by counting colony forming units in liquid cultures with the same optical density) compared with isogenic cells expressing the wild-type protein under non-selective conditions. We speculate that these fitness costs, in combination with the prevalence of other amikacin-modifying enzymes, hinder the evolution of APH(3′)-IIIa in clinical settings.

## Introduction

We strive to understand how enzymes originate and evolve. A better understanding of the rules that govern these adaptive processes would fill a knowledge gap in the Darwinian Paradigm, and guide protein engineers toward more efficient design algorithms. We have learned much from previous laboratory evolution experiments. Evolutionary theory suggests that new biological systems are products of contingency, when “a feature evolved long ago for a different use has fortuitously permitted survival during a sudden and unpredictable change in rules” [1]. At the molecular level, an enzyme that originally evolved to catalyze the conversion of a particular substrate into a particular product might also react similarly with other substrates (substrate ambiguity) or accelerate a different chemical transformation (catalytic promiscuity). These weak secondary functions could serve as seeds for subsequent evolutionary innovation.

Studies of individual enzymes *in vitro* showed that wild-type enzymes could be multi-functional [2], [3]. Pioneering directed evolution experiments showed that weak secondary activities can be physiologically relevant and evolvable [4], [5], [6]. In general, though with notable exceptions [7], [8], [9], [10], [11] artificially evolved enzymes are not as specific or efficient as their respective wild-type ancestors. Why does directed evolution usually fall short of the natural process? This question is not easily addressed [12], even after 20 years of directed protein evolution experiments [4], [13]. Protein engineers value speed, so they may be inclined to impose exceedingly stringent selections upon small populations for a relatively small number of generations. We also suspect that laboratory selection conditions are more uni-dimensional than those in the wild.

The TEM-1 beta-lactamase catalyzes the hydrolysis of beta-lactam antibiotics. It readily evolves, *in vitro* and *in situ*, to recognize cephalosporin antibiotics, so it serves as a model system for studies of protein evolution [4], [14], [15], [16], [17]. The aminoglycoside phosphotransferases (APH) also present attractive but under-utilized systems for evolutionary studies, as they diverged in nature to recognize and inactivate a wide range of commercially available, clinically relevant antibiotics [18]. APHs are expressed in the cytoplasm, unlike beta-lactamase, which is secreted into the periplasm where its activity can benefit other cells. Previous workers have shown that double mutations in one family member, aminoglycoside phosphotransferase (2′′)-IIa, could increase the resistance of host cells to amikacin and isepamecin eight-fold relative to the wild-type (as measured by differences minimum inhibitory concentrations) [19]. Changes of that magnitude are clinically relevant, but we believe that quantitatively larger adaptations are possible in the laboratory and that such experiments would be informative.

We chose aminoglycoside phosphotransferase (3′)-IIIa, or APH(3′)-IIIa, for our studies. It is broader in specificity than is APH(2′′)-IIa [18], and is therefore potentially more evolvable. APH(3′)-IIIa is well characterized [20], [21], [22], and was the first APH to be crystallized [23], so the biochemical mechanisms of mutations that accumulate during directed evolution can be inferred. We show here that the enzyme readily evolves to recognize amikacin, which is used to treat multidrug resistant gram negative pathogens, and that it is relatively easy to purify, store and assay *in vitro*. We also show that some mutations that are beneficial under selections are associated with significant fitness costs under non-selective conditions.

## Materials and Methods

### Chemicals and Reagents

Restriction enzymes (EcoRI, SalI, NdeI, HindIII), molecular biology enzymes (T4 DNA ligase), and reagents (1 kb DNA ladder) were from New England Biolabs (Ipswitch, MA). Taq DNA polymerase was over-expressed in *E. coli* and partially purified via heat treatment and dialysis [24]. The dNTPs were from Roche Applied Science (Indianapolis, IN). DNA purification kits (for plasmid, PCR and gel extraction) were from Qiagen (Valencia, CA). IPTG was from Gold Biotechnology (St. Louis, MO). Antibiotics (amikacin, ampicillin, chloramphenicol, kanamycin), buffer components (Tris-HCl), salts (KCl, MgCl_2_, MnCl_2,_ imidazole), assay enzymes (pyruvate kinase, lactate dehydrogenase), and substrates (adenosine triphosphate, nicotinamide adenine dinucleotide, phosphoenolpyruvic acid) were from Sigma-Aldrich (St. Louis, MO). DNA oligonucleotides were synthesized by Integrated DNA Technologies (Coralville, IA). Lonza Seakem LE agarose and Difco granulated agar were from Thermo Fisher Scientific (Waltham, MA). EMD Millipore Luria Broth was from VWR (Radnor, PA). Acrylamide for SDS gels was from Bio-Rad (Hercules, CA).

### Mutagenesis and Cloning

The *aph(3′)-IIIa* gene in the pBAV1K plasmid [25] was converted into a “BioBrick” (DNA sequence flanked by unique standard restriction sites [26]) by PCR amplification using primers 1 and 2 (Table 1). The product was subsequently recombined into the multiple cloning site of the pQBAV3c vector via overlap extension PCR cloning [25], [27]. Outside primers 3 and 4 were subsequently used to introduce random mutations in error prone PCRs (standard conditions with an extra 0.2 mM dCTP, 0.2 mM dTTP, 1.2 mM MgCl_2_ and 0.125 mM MnCl_2_, as previously described [28]), or to randomly recombine alleles in staggered extension process PCR (80 cycles with ultra-short 15 second extensions at sub-maximal 60°C temperatures) [29]. Outside primers 3 and 4 were used in combination with internal primers 5 and 6 in two separate PCRs (one with primers 3 and 6, the other with 4 and 5), followed by an overlap extension PCR (using primers 3 and 4), to introduce the D190A mutation into APH(3′)-IIIa. After each of these PCR reactions, restriction enzymes EcoRI and SpeI were used to clone the amplification products back into pQBAV3c.

**Table 1:** Primers used in this study.

Primer	Name	Sequence
1	KAN_STDBB_F	ggaattcgcggccgcttctagagaaattctatcataattgtggtttcaa
2	KAN_STDBB_R	ctgcagcggccgctactagtattattaaaacaattcatccagtaaaatataa
3	pQBAV3_MCS_−48_ecori_F	gcaaacaaaccaccgctggtagcg
4	pQBAV3_MCS_+278_psti_R	gccgtaatatccagctgaacggtctggttatag
5	aph_D190A_for	cttgtcttttcccacggcgccctgggagac
6	aph_D190A_rev	gtctcccagggcgccgtgggaaaagacaag
7	aph_pet28_F_ndeI_nhis	ggggtatctttaaatactgtagaaaagaggaaggaaataacatatggct
8	aph_pet28_R_hindIII_nhis	gtctgcagcggccgctactaaagcttttaaaacaattcatc

We wanted to over-express APH(3′)-IIIa with an N-terminal his_6_-tag, but did not want to use an expression system that produced its own aminoglycoside phosphotransferase. The *aph(3′)-Ia* gene of pET28a+ (Novagen) was thus replaced with the *bla* gene encoding TEM-1 beta-lactamase gene from pET20b+ using restriction enzymes AlwNI and XhoI. The *aph(3′)-IIIa* gene was PCR amplified with primers 7 and 8. The product was cloned into the modified pET28 vector using restriction enzymes NdeI and HindIII restriction sites, thereby fusing the *aph(3′)-IIIa* gene to sequence encoding a his_6_-tag on the N-terminus of the protein.

### Genetic Selection of Aph(3′)-IIIa Variants that Confer Decreased Amikacin Susceptibility

*E. coli* InvαF’ carrying the *aph(3′)-IIIa*-pQBAV3c plasmid were spread on LB agar plates with varying concentrations of amikacin after each round of selection. In the first round, 10,000 colonies were spread on LB agar supplemented with 18 micrograms/mL amikacin (“wild-type MIC”). Colonies that formed were then grown to saturation in liquid LB supplemented with chloramphenicol, diluted 10^−5^-fold; 50 microliters were spread on 10×LB plates containing 22, 26, 30, 35, 40, 45, or 50 micrograms/mL amikacin. In the second round, 10,000 colonies were spread on LB supplemented with 80 micrograms/mL amikacin. Colonies that formed were subsequently restruck as above on LB plates containing 160, 170, 180, 200 micrograms/mL amikacin. In the third round, colonies were selected on LB supplemented with 220 micrograms/mL amikacin, and those that formed were then diluted 5×10^−4^-fold and restruck on LB plates containing 250, 280, 310, 340, 370, 400, 425, 450, 500, 550, 600, 650, 700, 750, 800, 825, 850, 875, 900, 950, 1000, 1050, 1100, 1150, 1200, 2000 micrograms/mL amikacin. After the fourth round, colonies were spread (at approximately 1000 CFU/plate on 10 plates) on LB with 1200 micrograms/mL amikacin. The reported susceptibility of each mutant (Table 2) is the minimum concentration at which it was unable to form any colonies.

**Table 2:** Aph(3′)-IIIa sequences and minimum inhibitory concentrations (round 1).

Round ofMutagenesis	Mutant	Missense Mutations	SilentMutations	Non-Coding Mutations	Amikacin MIC (µg/mL)
1	1	**H78**Y, **V96A**		n.d.	30
1	2	**N38**D			26
1	3	I254M	**R211**	−192(A-G), −154(A-G), −135(T-C)	26
1	4	**Q236R**		−51(A-G)	30
1	5	**E9K**, **K12R**, **L136I**		−137(T-A), −20(A-G)	30
1	6	**K12E**	D261	−134(A-G), −53(T-G)	35
1	7	**D193N**		−134(A-G)	35
1	8	**V96I**, **K248E**		n.d.	30
1	9	F79Y, **Q236R**	**D167**	−174(T-C)	30
1	10			−113(T-C), −7(A-G)	30
1	11	M1V	G69	−177(T-C), −162(T-G), −157(T-C), −75(A-T)	26
1	12	I258T			30
1	13	Y102C	E235	−31(A-T)	30
1	14	D167G		−31(A-G)	30
1	15	E16G, **K255R**		−182(T-A)	26
1	16	K21E, **V198**A	L244	−161(C-T)	26
1	17			−35(T-C)	26
1	18	**S194R**		−86(A-G)	35
1	19	**L136I**	E80		26
1	20	T177A, D231G, **K248E**	**S27**, L259	−133(A-T), −112(T-G), −35(T-C)	26
1	21	**I40T**	**L10**		26
1	22		**L41**	−46(T-C)	30
1	23	E15K	L140	n.d.	26
1	24	**E160D**		−188(T-C), −29(T-C)	30
1	25		E234	−58(T-C)	26
1	26	E103G	L175, V185	−35(T-A)	35
1	27	**I112V**, **V198**M		−199(A-G), −58(T-C)	45
1	28			−77(G-A), −65 (G-C)	30
1	29	E181G	K21		30
1	30	**E9G**, K179R, **K255R**	P28	−23(A-G)	30
1	31		**E9**, D153	−142(A-T), −67(T-A)	30
1	32	D104V		−59(T-C), −49(C-T), −26(T-C)	26
1	33	**I112V**, E161G	**E234**	−21(A-G)	26
1	34	**H78**T, A152V	**E24**, **L41**	−6(T-A)	50
1	35	**N38**S	T55, **D144**	−132(A-G)	26
1	36	G36R, **E234G**, **K255E**	D190	−150(T-C), −98(T-C), −58(T-C)	26
1	37		**R211**	−35(T-C)	26
1	38		D94	n.d.	30
1	39			−105(T-C)	30
1	40		D137	−150(T-C), −52(T-C)	26
1	41	A2T	K43	−185(T-C), −181(G-A),−123(A-T), −104(T-C)	26
1	42		K253, E262	−74(T-C)	26
1	43			−31(A-G)	30

Recurring mutations are written in **bold** text.

### Purification of APH(3′)-IIIa Enzyme and Mutants

*E. coli* BL21(DE3) carrying the modified, beta-lactamase producing pET28 plasmid encoding *his*_6_-*aph(3′)-IIIa* were propagated to saturation overnight in a 4 mL culture of LB broth containing ampicillin. The starter culture was then added to a 400 mL culture of LB-ampicillin broth and grown for 4.5 hours at 37°C. At mid-log phase, protein expression was induced overnight by the addition 1 mM IPTG. The preparation and purification of was performed at 4°C. The 400 mL of the fully grown cultures were collected by centrifugation at 1540×*g* for 10 minutes. The cells were resuspended in 30 mL of 50 mM Tris, pH 7.5 and sonicated on ice for 5 minutes (5 cycles of 30 seconds on/off sonication at 6 watts by a Misonix Sonicator 3000). The cell lysate was eliminated by centrifugation at 20,100×*g* for 40 minutes, and the supernatant was saved. A his-trap column connected to an AKTA purifier (GE Healthcare) was washed with buffer B (50 mM Tris pH 7.5, 400 mM imidazole) and then equilibrated with buffer A (50 mM Tris pH 7.5) before the supernatant was run through the column. The column was washed with buffer A before applying a step gradient of buffer B to elute the his_6_-APH(3′)-IIIa enzyme. Fractions containing the enzyme were dialyzed (Spectra #132720– MWCO: 3,500) in 50 mM Tris pH 7.5 and analyzed by SDS-polyacrylamide gel electrophoresis to verify purification.

### Enzyme Assays

A coupled assay with pyruvate kinase/lactate dehydrogenase was used to measure the formation of ADP [30], [31]. Assay buffer (975 microliters of 50 mM Tris pH 7.6, 40 mM KCl, 10 mM MgCl_2_, 0.25 mg/mL NADH, 2.5 mM PEP and 1 mM ATP in a quartz cuvette, Fisher #14-385-914A) was mixed with 5 microliters of pyruvate kinase/lactate dehydrogenase (600–1000 u/mL PK, 900–1400 u/mL LDH) and 10 microliters of the appropriate concentration of aminoglycoside, then incubated at 37°C for 15 minutes. The reaction was initiated with the addition of 10 microliters of stock solution (1 micromolar) of his_6_-APH(3′)-IIIa. Absorbance at 340 nm, indicating oxidation of NADH, was monitored with a Shimadzu UV-1601 spectrophotometer for 5 minutes. The rate of oxidation of NADH was determined using its extinction coefficient (6220 mol^−1^ cm^−1^) [32]. This rate was converted into the initial velocity of the APH(3′)-IIIa-catalyzed phosphotransfer reaction. The data were fit to standard kinetic models by the methods of least squares using Kaleidagraph v3.5 (Synergy Software).
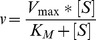


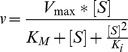



### Fitness Assays

*E. coli* InvαF’ carrying the wild-type or variant *aph(3′)-IIIa*-pQBAV3c plasmids were grown overnight to saturation in 2 mL LB broth with 34 micrograms/mL chloramphenicol or 50 micrograms/mL kanamycin. A 200 microliter sample of each culture was transferred into the well of a clear, flat bottom 96 well plate (VWR # 62409-068), and the optical density at 600 nm of each culture was measured in a plate reader (Biotek Synergy 2). Each culture was serially diluted three times, each time with 20 microliters of the sample being added to 980 microliters of LB broth; 50 microliters of the final serial dilution were spread on LB plates containing 34 micrograms/mL chloramphenicol or 50 micrograms/mL kanamycin. Plates were incubated at 37°C overnight, and the visible colonies were counted.

*E. coli* InvαF’ containing the *aph(3′)-IIIa*-pQBAV3c plasmid with was grown overnight to saturation in 2 mL LB broth with 34 micrograms/mL chloramphenicol or 50 micrograms/mL kanamycin. Each culture was serially diluted twice, each time with 20 microliters of the sample being added to 980 microliters of LB broth. A 200 microliter sample of each culture was transferred into a clear, flat bottom 96 well plate. The plate was agitated at a medium speed at 37°C for 24 hrs in a Biotek Synergy2 microtiter plate reader; the optical density at 600 nm was measured every 30 minutes.

## Results

### APH(3′)-IIIa Readily Adapts to Amikacin

APH(3′)-IIIa is well-suited for our study of adaptive enzyme evolution because, as we explained above, its weak activities against a range of antibiotics enables facile selections in *Escherichia coli* [25]. We chose amikacin as the “novel” substrate because it is nearly identical in structure to its “native” (most reactive) substrate, kanamycin, from which it is chemically synthesized [33]. The *aph(3′)-IIIa* gene was amplified and randomly mutated in an error-prone PCR; the resulting library cloned into pQBAV3c [27], which also encodes chloramphenicol acetyltransferase. *E. coli* strain InvαF’ was transformed with the plasmid-borne library; 10,000 colony-forming units were spread on 10×(100×15 mm) Petri dishes containing LB agar supplemented with chloramphenicol and 18 micrograms/mL amikacin. Isogenic control cells transformed with the ancestral *aph(3′)-IIIa*-pQBAV3c plasmid do not grow under these conditions, but 43 colonies formed among the approximately 10,000 that expressed mutant APH(3′)-IIIa proteins. The 43 selected *aph(3′)-IIIa* alleles were sequenced (Table 2); most contained just 1 or 2 nucleotide mutations in the open reading frame (ORF) (27/43 mutants). Nearly all alleles also contained mutations in the upstream 5′ region, including those without ORF mutations. Each mutant was restruck on fresh plates containing higher concentrations of amikacin (22–50 micrograms/mL) in order to measure the minimum inhibitory concentration (MIC, Table 2).

The 43 variant *aph(3′)-IIIa*-pQBAV3c plasmids were pooled, along with the ancestral plasmid, and used as templates for staggered extension process (StEP) recombination [29]. The resulting recombinant library was ligated back into the pQBAV3c plasmid. *E. coli* InvαF’ was transformed with the library and spread on LB agar plates supplemented with 80 micrograms/mL amikacin. Seven colonies out of approximately 10,000 formed under these more stringent conditions (Table 3). We sequenced these *aph(3′)-IIIa* alleles and found that this small population was dominated by two new mutations (I40T and D193N) and two others (S194R and K255R) that were selected in the first round. Most of the selected mutants unexpectedly contained only single amino acid changes (4/7 mutants). It is possible that the increase in amikacin resistance of these mutants in this round was due to mutations outside of the ORF; mutants 2.4 and 2.5 had the same ORF mutation as 1.18 but additional non-ORF mutations (most notably −58(T-C) and −35(T-C)). It is also possible that the amikacin resistance can increase from the elimination of slightly deleterious mutations; mutant 2.3 shares a mutation with mutants 1.27 and 1.33, but is missing some mutations unique to them.

**Table 3:** Aph(3′)-IIIa sequences and minimum inhibitory concentrations (rounds 2–4).

Round of Mutagenesis	Mutant	Missense Mutations	Silent Mutations	Non-Coding Mutations	Amikacin MIC (µg/mL)
2	1	**I40T**, **K255R**		−35(T-A)	160
2	2	**I40T**, **S194R**, **K255R**	**L10**, **V73**	−31(A-G)	200
2	3	**I112V**		−58(T-C), −31(A-G)	160
2	4	**S194R**		−86(A-G), −58(T-C)	160
2	5	**S194R**		−35(T-C), −24(T-C)	160
2	6	**D193N**		−92(A-G), −77(G-A),−65(G-C)	170
2	7	**D193N**	**E24**	−31(A-G), −6(T-A)	160
3	1	K3R, I6M, **I40T**, **D144G**, **E160G**, **K176R**, **S194R**, **I196F**, **K255R**	**E9**, **L10**, **S27**, **V73**, V96	−185(T-C), −172(A-G), −31(A-G)	280
3	2	**I112V**, **S194R**		−58(T-C), −32(A-G)	700
3	3	**E24V**, **I40V**, **S194R**	E68, L140	−90(T-C), −87(A-T),−86(A-G0, −58(T-C)	1200
3	4	K11N, **I40T**, **R120K**, **C156R**, **S194R**, **K255R**	**L10**, **V73**	−102(A-T), −31(A-G)	500
4	1	**E24V**, **I40T**, **R120K**, **C156R**, **K176R**, **S194R**, **I196F**, Y219H, **K255R**	**V73**, V76	−102(A-T), −31(A-G)	–

The seven selected *aph(3′)-IIIa* alleles were pooled, amplified, and mutated in an error-prone PCR. The mutated genes were cloned back into the pBAV3c plasmid. InvαF’ cells were transformed with the cloned library, and spread on LB agar plates supplemented with 220 micrograms/mL amikacin. Four colonies (out of approximately 10,000 transformants) formed under these selection conditions. DNA sequencing of the four associated alleles showed that each contained the S194R and three of the four mutants (3.1, 3.3 and 3.4) also had a mutation in the 40th residue, although there were two different mutations (I40V and I40T) at this position. The D193N mutation, carried by two mutants from the second round of evolution went extinct in the third.

**Figure 1:**
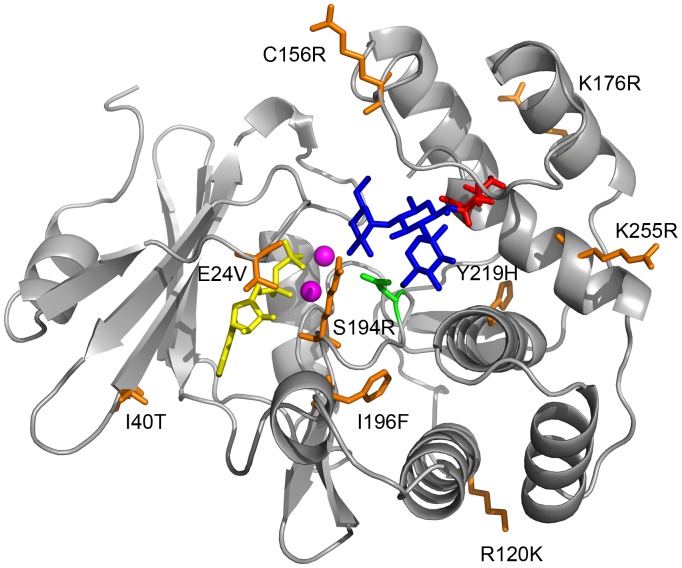
Model of artificially evolved aminoglycoside phosphotransferase (3′)-IIIa, based upon crystal structure (1L8T) of the wild-type enzyme [23] rendered in PyMOL. The putative beneficial mutations are colored orange, the catalytic D190 residue is green), adenosine diphosphate is yellow and magnesium is pink. Kanamycin is blue, while the “extra” moiety that differentiates amikacin from kanamycin is red.

The four selected plasmids, and their ancestor, which encodes the wild-type *aph(3′)-IIIa* gene, were pooled and used as templates for StEP recombination. The recombinant library was cloned and used to transform InvαF’. The transformants were challenged with LB agar supplemented with 1200 micrograms/mL amikacin. One colony (out of approximately 10,000 transformants) formed. The DNA sequence of mutant 4.1 (E24V, I40T, R120K, C156R, K176R, S194R, I196F, Y219H, K255R; Figure 1) showed the persistence of three mutations that appeared in the first two rounds, I40T, S194R, and K255R (Table 2). We speculate upon the biochemical mechanisms of these adaptations in the Discussion section (*vide infra*).

### The 4.1 Aph(3′)-IIIa Variant is as Catalytically Efficient as its Wild-type Progenitor

**Figure 2:**
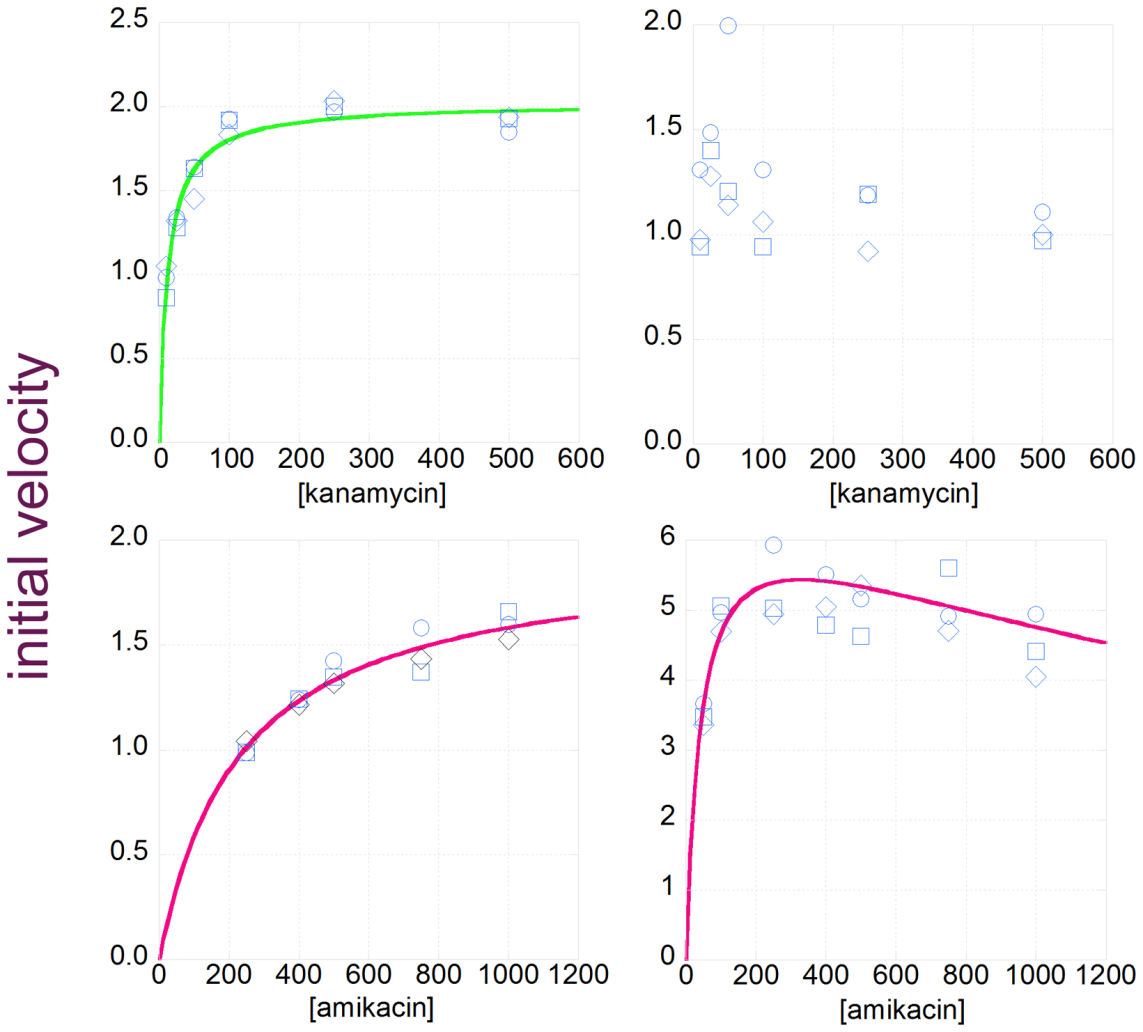
Michaelis-Menten plots of the wild-type and 4.1 APH(3′)-IIIa variants. The plots show the dependence of initial velocity upon substrate concentration for the following reactions: a) wild-type APH(3′)-IIIa with kanamycin, b) artificially evolved 4.1 variant with kanamycin, c) wild-type APH(3′)-IIIa with amikacin, and d) 4.1 variant with amikacin. Substrate concentration is in units of micromolar and reaction velocity is in moles of substrate/moles of enzyme/second. Each series of reactions was conducted in triplicate. The average initial velocity values were fit to the Michaelis-Menten equation (a, c) or a simple substrate inhibition model (d) as described in the Methods; the derived kinetic parameters are presented in Table 4.

The wild-type and 4.1 *aph(3′)-IIIa* alleles were subcloned into a modified version of pET28a+ that encodes the TEM-1 beta-lactamase in place of the usual *aph(3′)-Ia* [18]. The two recombinant plasmids were separately used to transform the *E. coli* production strain BL21(DE3). The proteins were over-expressed and purified by virtue of N-terminal hexahistidine tags encoded by the pET28a+ vector. The kinetic parameters of the two enzyme variants in reactions with the “native” substrate (kanamycin) and “novel” substrate (amikacin) were measured with a coupled pyruvate kinase/lactate dehydrogenase assay (Table 4, Figure 2). The *K*_M_ and *k*_cat_ of the his-tagged enzyme, his_6_-APH(3′)-IIIa in reactions with kanamycin and amikacin were similar to the published values of the native untagged form [21]. The evolved his_6_-4.1 APH(3′)-IIIa exhibited substantially higher *k*_cat_ and lower *K*_M_ in reactions with amikacin (and detectable substrate inhibition, *K*i >2 mM), when compared to the his_6_-wildtype APH(3′)-IIIa. The second order rate constant (*k*_cat_/*K*_M_) was similar to that of the wild-type enzyme in reactions with kanamycin. The evolved enzyme retains some catalytic activity in reactions with kanamycin, but its steady state enzyme kinetics could not be fit to the Michaelis-Menten equation or to any substrate inhibition model [34]. The interaction between his_6_-4.1 APH(3′)-IIIa and kanamycin is likely complex; we speculate that multiple non-productive binding modes compete.

**Table 4:** Kinetic Parameters of the wild-type and evolved 4.1 APH(3′)-IIIa.

	wild-type				4.1			
Substrate	*k*_cat_ (s^−1^)	*K_m_* (µM)	*K*_i_ (mM)	*k*_cat_/*K_m_* (M^−1^ s^−1^)	*k*_cat_ (s^−1^)	*K_m_* (µM)	*K*_i_ (mM)	*k*_cat_/*K_m_* (M^−1^ s^−1^)
kanamycin	2.02±0.05	12.1±1.8	n.d.^a^	1.67×10^5^	n.f.^b^	n.f.^b^	n.f.^b^	n.f.^b^
amikacin	1.95±0.14	232±41	n.d.^a^	8.42×10^3^	6.86±0.8	42.9±13	2.50±1.47	1.60×10^5^

a n.d. = not detected.

b n.f. = not fit.

### Fitness Costs of Beneficial Mutations

We noticed during our directed evolution experiments that some of the selected clones formed fewer colonies than did the isogenic ancestral strain. Fresh InvαF’ cells were transformed with *aph(3′)-IIIa*-pQBAV3c plasmids encoding the wild-type, evolved 2.3, 3.1 or 4.1 alleles (or pBC or pACYC Duet as controls). The transformants were propagated in parallel under non-selective conditions (liquid LB supplemented with 34 micrograms/mL chloramphenicol or 50 micrograms/mL kanamycin). The optical density (600 nm) of each culture was measured; the cultures were serially diluted, then spread on LB agar plates supplemented with either chloramphenicol or kanamycin. We observed significant and reproducible differences among isogenic transformants in growth rates (during log phase in liquid culture) and colony forming ability (Table 5).

**Table 5:** Fitness of transformed isogenic *Escherichia coli*.

Plasmid	colony formation chloramphenicol(cfu/mL/OD_600_)	colony formation kanamycin(cfu/mL/OD_600_)	growth rate chloramphenicol(OD_600_/min)
pBC (*lacZ*α)	2.7×10^8^±1.3×10^8^	0	0.0039±0.00066
pACYC Duet (lower copy #,*lacI*)	7.3×10^8^±1.8×10^8^	0	0.0051±0.00059
WT aph(3′)-IIIa-pQBAV3c	4.9×10^8^±1.7×10^8^	5.2×10^8^±5.8×10^7^	0.0026±0.00053
D190A aph(3′)-IIIa-pQBAV3c	6.2×10^7^±2.4×10^7^	0	0.0016±0.0011
2.3 aph(3′)-IIIa-pQBAV3c	8.0×10^7^±1.0×10^7^	6.3×10^7^±2.1×10^7^	0.0024±0.0012
3.1 aph(3′)-IIIa-pQBAV3c	4.6×10^8^±4.4×10^7^	3.8×10^8^±1.7×10^7^	0.0019±0.0017
4.1 aph(3′)-IIIa-pQBAV3c	1.2×10^8^±2.5×10^7^	9.1×10^7^±1.5×10^7^	0.0017±0.00020
4.1+ D190A aph(3′)-IIIa-pQBAV3c	7.3×10^8^±2.2×10^7^	0	0.015±0.000099

Two to eight-fold decreases in fecundity would almost certainly be decisive in nature, so we consider them worthy of further study. We first wondered whether the proliferation of untransformed cells in liquid culture, those that absorb light at OD_600_ but fail to form colonies on agar plates containing chloramphenicol, could explain the observed differences in fitness. If mutations in the *aph(3′)-IIIa* gene could affect plasmid stability, a disparity in colony forming ability would be revealed by growing *E. coli* InvαF’ containing pQBAV3c on plates both with and without selection for plasmid retention. We observed little or no plasmid loss in cells expressing the wild-type or 4.1 variants of APH(3′)-IIIa (Table 6). This result suggests that the fitness differences we observed (Table 5) are consequences of sequence differences in the *aph(3′)-IIIa* alleles themselves, rather than of indirect effects upon plasmid stability.

**Table 6:** Stability of ancestral and evolved *aph(3′)-IIIa*-pQBAV3c plasmids.

	No Plasmid	wild-type APH(3′)-IIIa	evolved 4.1 APH(3′)-IIIa
CFU/OD/mL (LB only)	1.41±0.19×10^8^	1.82±0.31×10^8^	9.71±2.41×10^7^
CFU/OD/mL (Chloramphenicol)	0	1.75±0.24×10^8^	8.93±2.53×10^7^
Plasmid Retention	–	97±9.0%	92±8.4%

We wondered whether the fitness costs correlate with improvements in activity against amikacin. We already have circumstantial evidence against this hypothesis. The intermediate 2.3 *aph(3′)-IIIa*-pQBAV3c imparts decreased fitness (relative to isogenic cells carrying its wild-type *aph(3′)-IIIa*-pQBAV3c ancestor), while mutant 3.1 did not (Table 5). Furthermore, the fitness associated with that APH(3′)-IIIa variants that we tested was unaffected by kanamycin (Table 5), suggesting that active-site occupancy apparently does not affect the fitness. To investigate our hypothesis more decisively, a single point mutation was made in the 190th residue of the wild-type and 4.1 APH(3′)-IIIa variants, changing the catalytic aspartic acid into alanine. This well characterized mutation has no significant effect on the structure or stability of APH(3′)-IIIa, but it abrogates detectable catalytic activity [20]. As expected, *E. coli* InvαF’ expressing the D190A or 4.1+ D190A APH(3′)-IIIa failed to grow in LB medium supplemented with kanamycin (Table 5) or amikacin (data not shown).

To our surprise, however, the fitness effects of the D190A mutation were context-dependent. Cells expressing the D190A APH(3′)-IIIa protein were significantly less fit than those expressing the wild-type protein under non-selective conditions. In contrast, cells expressing the 4.1+ D190A APH(3′)-IIIa were much fitter than isogenic cells expressing the 4.1 variant (Table 5). We don’t know whether the D190A and 4.1 proteins debilitate fitness through different biochemical mechanisms, or whether these protein variants act through a common non-catalytic mechanism that is sensitive to epistatic interactions among mutations. Many have observed that chromosomal mutations that impart resistance to other antibiotics come with a fitness cost. The population biology of these phenomena may be broadly similar [35], [36], [37], [38], [39], but the biochemical mechanisms are almost certainly idiosyncratic.

## Discussion

### Structural Hypotheses

The evolved 4.1 APH(3′)-IIIa contained nine amino acid replacements relative to its wild-type ancestor: E24V, I40T, R120K, C156R, K176R, S194R, I196F, Y219H and K255R. All but one, Y219H, first appeared in previous rounds of selection, suggesting that they are beneficial with respect to amikacin recognition. Two, namely I40T and S194R, are apparently beneficial in isolation. Amikacin is structurally identical to kanamycin, except that it contains an extra bulky modification (represented as red sticks in Figure 1) that creates a steric clash with the dynamic aminoglycoside binding loop (residues 147–170), at least in its kanamycin-binding conformation. The E157, N158 and E160 residues in that loop form hydrogen bonds with the amine groups in middle saccharide ring of kanamycin (including the one modified in amikacin) [23]. We therefore hypothesize that the C156R mutation increases the conformational flexibility of the loop, enabling it to accommodate amikacin. Two other mutations, Y219H and K255R, occur in alpha-helices that interact with the binding loop, and could therefore influence its conformation.

The D190 residue, which we mutated, is in different active site loop (residues 188–195), and forms a hydrogen bond with the hydroxyl group that the enzyme later phosphorylates [23]. The S194R and I196F mutations in that loop could increase its conformational flexibility, so that amikacin can bind in an orientation different than that of kanamycin. The E24 residue, located in yet another active-site loop (residues 22–29), forms a hydrogen bond with neomycin B, but not with kanamycin. We hypothesize that the E24V mutation destabilizes an unproductive binding mode. Other mutations in the evolved 4.1 APH(3′)-IIIa, namely I40T, I120K and K176R, occurred in residues more distant from the active-site, so it is more difficult to speculate about their effects upon amikacin recognition.

Most wild-type proteins are only marginally stable. Most amino acid changes are destabilizing, so the evolvability of most proteins is limited by conformational stability [40]. Mutations that alter the molecular recognition properties of an enzyme are particularly likely to be destabilizing [41]. Two active-site mutations that we observed, S194R and I196F, probably destabilize the active conformation by introducing new steric clashes with adjacent residues. The other seven amino acid changes occurred in surface residues, so their effects upon thermostability, if any, are less obvious. Global suppressor mutations, such as M182T in the TEM-1 beta-lactamase [16], can offset the destabilizing effects of beneficial mutations, but we made no deliberate effort to select for such mutations [42] nor did we see evidence for any. These hypotheses could be tested by calorimetric measurement of the thermodynamic parameters of the wild-type and mutant proteins.

### Clinical Implications

The *aph(3′)-IIIa* gene has appeared in clinical samples tested for resistance to kanamycin and other aminoglycosides [43], [44], but not amikacin [45], [46], [47]. We showed here, however, that four rounds of mutation and selection were sufficient to direct the evolution of an APH(3′)-IIIa variant (4.1) that conferred resistance to 60 times higher concentrations of amikacin than did the wild-type. The MIC exceeded 1200 micrograms/mL, substantially higher than the highest serum level (35 micrograms/mL) recommended in humans [48]. The aminoglycoside modifying enzymes diverged to adapt to different aminoglycosides in nature [49], but no mutants of these enzymes have been identified in clinical isolates [19]. It is tempting to speculate that the fitness costs observed in our study, in combination with the plethora of existing aminoglycoside modifying enzymes that efficiently confer resistance to amikacin, including AAC(6′)-1ad, ANT(4′)-II, and APH(3′)-VI [45], [46], [47] collude to prevent the adaptive evolution of APH(3′)-III in the clinic.
